# Heterogeneous Frustrated Lewis Pair Catalysts: Rational Structure Design and Mechanistic Elucidation Based on Intrinsic Properties of Supports

**DOI:** 10.1021/acs.accounts.4c00683

**Published:** 2025-01-28

**Authors:** Jiasi Li, Guangchao Li, Shik Chi Edman Tsang

**Affiliations:** † The Wolfson Catalysis Centre, Department of Chemistry, University of Oxford, Oxford OX1 3QR, U.K.; ‡ Crystallography Group, Diamond Light Source, Diamond House, Harwell Science and Innovation Campus, Fermi Avenue, Didcot OX11 0DE, U.K.; § Department of Applied Biology and Chemical Technology, The Hong Kong Polytechnic University, Hong Kong 999077, China

## Abstract

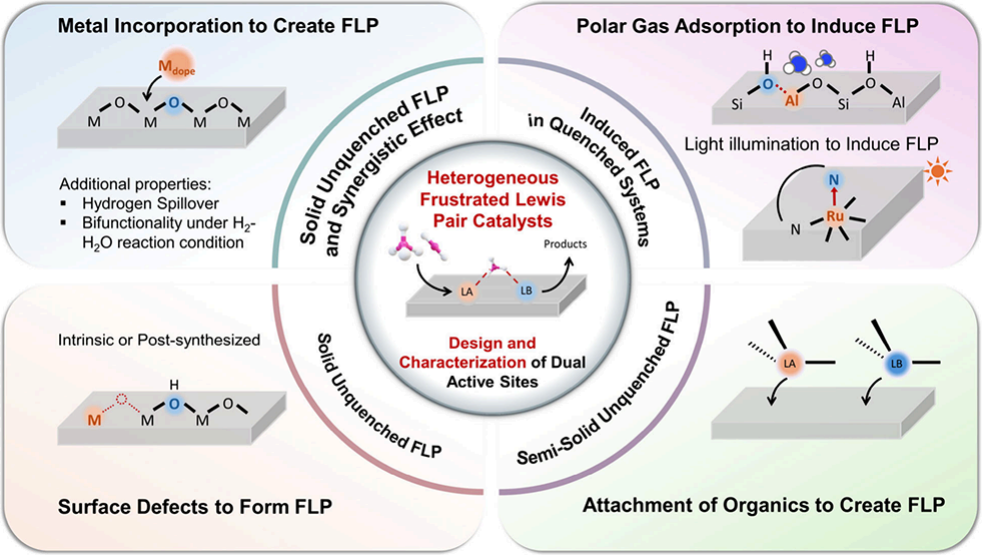

The discovery of reversible hydrogenation using metal-free phosphoborate species in 2006 marked the official advent of frustrated Lewis pair (FLP) chemistry. This breakthrough revolutionized homogeneous catalysis approaches and paved the way for innovative catalytic strategies. The unique reactivity of FLPs is attributed to the Lewis base (LB) and Lewis acid (LA) sites either in spatial separation or in equilibrium, which actively react with molecules. Since 2010, heterogeneous FLP catalysts have gained increasing attention for their ability to enhance catalytic performance through tailored surface designs and improved recyclability, making them promising for industrial applications. Over the past 5 years, our group has focused on investigating and strategically modifying various types of solid catalysts with FLPs that are unique from classic solid FLPs. We have explored systematic characterization techniques to unravel the underlying mechanisms between the active sites and reactants. Additionally, we have demonstrated the critical role of catalysts’ intrinsic electronic and geometric properties in promoting FLP formation and stimulating synergistic effects. The characterization of FLP catalysts has been greatly enhanced by the use of advanced techniques such as synchrotron X-ray diffraction, neutron powder diffraction, X-ray photoelectron spectroscopy, extended X-ray absorption fine structure, elemental mapping in scanning transmission electron microscopy, electron paramagnetic resonance spectroscopy, diffuse-reflectance infrared Fourier transform spectroscopy, and solid-state nuclear magnetic resonance spectroscopy. These techniques have provided deeper insights into the structural and electronic properties of FLP systems for the future design of catalysts.

Understanding electron distribution in the overlapping orbitals of LA and LB pairs is essential for inducing FLPs in operando in heterogeneous catalysts through target electron reallocation by external stimuli. For instance, in silicoaluminophosphate-type zeolites with weak orbital overlap, the adsorption of polar gas molecules leads to heterolytic cleavage of the Al^δ+^–O^δ−^ bond, creating unquenched LA–LB pairs. In a Ru-doped metal–organic framework, the Ru–N bond can be polarized through metal–ligand charge transfer under light, forming Ru^+^–N^–^ pairs. This activation of FLP sites from the framework represents a groundbreaking innovation that expands the catalytic potential of existing materials. For catalysts already employing FLP chemistry to dynamically generate products from substrates, a complete mechanistic interpretation requires a thorough examination of the surface electronic properties and the surrounding environment. The hydrogen spillover ability on the Ru-doped FLP surfaces improves conversion efficiency by suppressing hydrogen poisoning at metal sites. In situ H_2_–H_2_O conditions enable the production of organic chemicals with excellent activity and selectivity by creating new bifunctional sites via FLP chemistry. By highlighting the novel FLP systems featuring FLP induction and synergistic effects and the selection of advanced characterization techniques to elucidate reaction mechanisms, we hope that this Account will offer innovative strategies for designing and characterizing FLP chemistry in heterogeneous catalysts to the research community.

## Key References

- LiG.; FooC.; YiX.; ChenW.; ZhaoP.; GaoP.; YoskamtornT.; XiaoY.; DayS.; TangC. C.; Induced Active Sites by Adsorbate in Zeotype Materials. J. Am. Chem. Soc.2021, 143 ( (23), ), 8761–877134076425 10.1021/jacs.1c03166.^1^*Induced frustrated Lewis pairs from Brønsted Lewis sites in SAPO-type materials upon adsorption of polar gas molecules was discovered and characterized for the first time.*
- NgB. K. Y.; ZhouZ.-J.; LiuT.-T.; YoskamtornT.; LiG.; WuT.-S.; SooY.-L.; WuX.-P.; TsangS. C. E.Photo-Induced Active Lewis Acid–Base Pairs in a Metal–Organic Framework for H_2_ Activation. J. Am. Chem. Soc.2023, 145 ( (35), ), 19312–1932037611205 10.1021/jacs.3c05244PMC10485891.^2^*Induced frustrated Lewis pairs via metal-to-ligand charge transfer activated by light were first observed in Ru/UiO-67-bpydc material.*
- WuS.; TsengK.-Y.; KatoR.; WuT.-S.; LargeA.; PengY.-K.; XiangW.; FangH.; MoJ.; WilkinsonI.; Rapid Interchangeable Hydrogen, Hydride, and Proton Species at the Interface of Transition Metal Atom on Oxide Surface. J. Am. Chem. Soc.2021, 143 ( (24), ), 9105–911234047552 10.1021/jacs.1c02859.^3^*Reversible hydrogen spillover via a frustrated Lewis pair Ru–O in Polar Ru-doped MgO(111) was first reported and characterized in the system, which advanced the design of hydrogenolysis types of catalysts.*
- DengQ.; LiX.; GaoR.; WangJ.; ZengZ.; ZouJ.-J.; DengS.; TsangS. C. E.Hydrogen-Catalyzed Acid Transformation for the Hydration of Alkenes and Epoxy Alkanes over Co–N Frustrated Lewis Pair Surfaces. J. Am. Chem. Soc.2021, 143 ( (50), ), 21294–2130134874721 10.1021/jacs.1c08259.^4^*H_2_-assisted hydration of alkenes and epoxy alkanes over Co–N surfaces via a H_2_-catalyzed acid–base transformation mechanism on Co–N frustrated Lewis pairs was reported for the first time.*

## 1 Introduction

### 1.1 Pioneering Research on Homogeneous Frustrated Lewis Pairs

**Figure 1:**
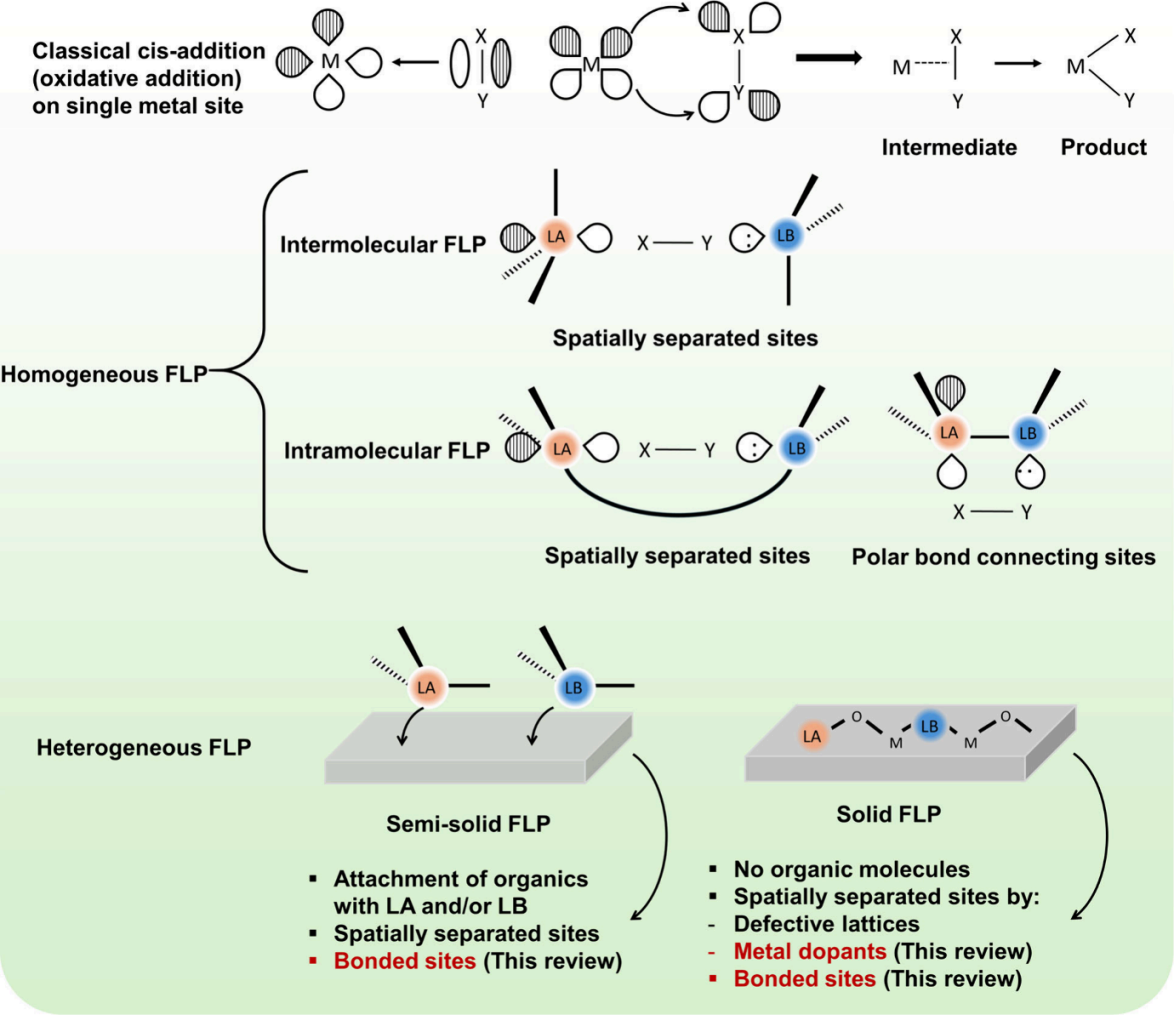
Overview of different FLP types in homogeneous and heterogeneous catalysts.

Frustrated Lewis pair (FLP) chemistry drives innovation in catalyst design, exploring the catalytic potential of elements across the periodic table (Figure 1). Building on earlier research in homogeneous catalysis, the FLP concept was first characterized by the Stephan group in 2006 with a metal-free phosphonium borate compound.^5^ In this system, steric constraints between the intramolecular Lewis acidic boron and Lewis basic phosphorus prevent dative bond formation, pioneering the research on reversible hydrogen activation reactions. Unlike the classical homolytic cleavage of hydrogen, which features concerted cis addition of H atoms on a single transition metal site via electron back-donation to the σ* bond,^6−10^ FLP systems achieve hydrogen cleavage through electron transfer (ET) between Lewis base (LB) and Lewis acid (LA) sites. Subsequent studies on P/B,^11−14^ N/B,^15^ and other systems formed by main-group elements as well as complex systems with reactive transition metals and ligands^16−19^ further refined the definition of FLP chemistry. Both intermolecular and separated intramolecular FLP sites exhibit activation activity; intramolecular systems with classical Lewis acid–base adducts in equilibrium and featuring polar bonds that retain donor and acceptor properties also meet the conditions for molecular activation. In all these systems, ET is involved. Based on these principles, carbene and enzyme chemistry are now also considered within the scope of FLP-type activation, with more interdisciplinary systems being investigated.^18,20^ Inspired by hydrocracking attempts using these catalysts, the application of homogeneous FLP chemistry has been broadened. Hydrogenation (or reduction) of organic molecules is the crucial application mediated by FLPs.^21^ Meanwhile, the abilities of FLPs to assist hydrogen transfer and dehydrogenation of organic molecules have also been reported.^18^ The electron density difference between sites allows for capturing and activating small molecules such as CO, CO_2_, and N_2_O in the H_2_ environment, which is useful for applications such as air purification.^22−24^ C–H bond activation can also be achieved via these FLP sites.^23,25^

### 1.2 Exploration of Heterogeneous FLPs

Since the 2010s, researchers have expanded their focus from homogeneous FLP catalysts to FLP chemistry in heterogeneous systems to improve product separation and facilitate large-scale commercial applications.^26,27^ In heterogeneous systems, controlled modifications based on the intrinsic rigidity of the surface sites on solid materials can increase the catalytic activity, stability, and chemical selectivity. Through attachments of organic molecules with acidic or basic properties on inert 2D materials or porous 3D supports, a transition from a homogeneous to heterogeneous catalyst is established. Examples include semisolid FLP catalysts such as Au coupled with Lewis bases like imines,^28^ Al/P or P/B compounds anchored to silica,^29,30^ and N/B anchored to MIL-101(Cr).^31^ Some solid materials feature intrinsic FLP systems due to their rigid surface topography and defects, even without organic adsorbates. These natural solid FLPs are commonly found in reducible metal oxides_,_ with CeO_2_ being a typical example.^32−35^ Benefiting from mild redox properties, the reduced metal center near oxygen vacancies acts as an LA and adjacent surface oxygen as an LB, and they are spatially separated from orbital mixing. AlOOH is another recent example, with deprotonated oxygen as the Lewis base and unsaturated Al^3+^ as the acid site.^36^ In other cases, defects can be introduced postsynthesis to create FLP systems. For instance, in 2011 Sautet et al.^37^ revisited the active sites of a highly effective catalyst, γ-Al_2_O_3_, for C–H bond activation of methane. Their experimental and density functional theory (DFT) studies revealed the formation of reactive acid–base pairs in (110) termination, between defective Al(III) and oxygen atoms adjacent to Al(IV), which adsorb water for surface hydroxylation.^38^ In addition to hydration, thermal treatment can also introduce FLPs, for instance, the activation of In_2_O_3–*x*_(OH)_*y*_ from In_2_(OH)_3_ for the reverse water-gas shift reaction.^39^ Metal incorporation is another explored field that can generate more oxygen vacancies^40,41^ or form new FLP systems with the basic sites from the support.^42,43^ Recent developments on heterogeneous FLPs are summarized in Table 1 to present a more comprehensive review of the systems.

**Table 1:** Brief Summary of Recent Developments in Heterogeneous FLP Chemistry in Different Systems

examples	types	FLP	applications	key evidence for FLP chemistry
boehmite (AlOOH) surfaces,^36^ CeO_2_,^45^ ZnIn_2_S_4_/In(OH)_3–*x*_ heterojunction^51^	intrinsic FLP sites (defects)	LA: unsaturated metal site	hydrogenation,^36,45^ photocatalytic CO_2_ reduction^51^	XPS, FTIR, DFT
		LB: O/OH sites adjacent to surface vacancy (e.g., Ov, OHv)		
wurtzite crystal surfaces, including GaN, ZnO, and AlP^52^	intrinsic FLP sites (defects)	LA: surface cations	activation of small molecules, e.g., H_2_, CH_4_, NH_3_, H_2_S, and PH_3_	DFT
		LB: surface anions		
Cs_2_CuBr_4_ perovskite quantum dots^53^	intrinsic FLP sites	LA: Cu central atom surrounded by Br atoms	CO_2_ photoreduction	in situ DRIFTS, DFT
		LB: sterically isolated Cs atoms		
mixed diblock copolymers^54^	intrinsic FLP sites	LA: 4-styryl-di(pentafluorophenyl)borane	CO_2_ conversion	NMR
		LB: 4-styryldimesitylphosphine		
nitrogen incorporated cerium oxide^55^	FLP sites (defects) generated by dopants	LA: unsaturated Ce site	photocatalytic CO_2_ reduction	XPS, DRIFTS, DFT
		LB: hydroxyl adjacent to surface O_v_		
carbon-encapsulated Ni/NiO_*x*_^56^	FLP sites (defects) generated by dopants	LA: V_Ni_–C	photothermal-assisted photocatalytic hydrogen production	DFT
		LB: V_O_–C		
porous CeO_2_ nanorods (Pt cluster/PN–CeO_2_),^32^ Pt_1_/CeO_2_ (SA)^57^	intrinsic FLP sites (defects) and metal sites (dual-active sites)	LA: unsaturated Ce site	reverse water-gas shift reaction^58^	XPS, DRIFTS, DFT, KIE for hydrogen spillover
		LB: oxygen (hydroxyl) adjacent to surface O_v_	nonoxidative coupling of methane^57^	
Ru-doped MgO,^43^ Ru/NH_2_-rGO,^59^ Cu/In_2_O_3_ (SA)^60^	between metal dopant and support	LA: metal dopant	hydrogenation	DRIFTS, EPR, isotopic labeling, DFT
		LB: oxygen from the support	co-conversion of CH_4_ and CO_2_^60^	
boron- and sulfur-codoped graphitic carbon nitride (g-C_3_N_4_)^61^	between dopant and support/intrinsic	LA: electron-deficient S atom	photocatalytic CO_2_ reduction	DFT
		LB: electron-rich N atom active site adjacent to a B atom		
polyoxometalate (POM)-based MOF^62^	between guest and support MOF	LA: coordination-defect metal nodes of MOF	hydrogenation	XAS, XPS, DRIFTS, DFT
		LB: surface oxygen atoms in POM		
Zr-based MOF^63^	between guest and support MOF (in situ formation of FLP)	LA: boron-functionalized linker	CO_2_ chemical fixation	XPS, NMR, DFT
		LB: amine substrate		
COF-TAPB-3P-COOH^64^	between anchored LB and support COF	LA: 3P-COOH from COF	hydrogenation	FTIR, DFT
		LB: Lewis base reacted with COF		
defective boron carbon nitride^65^	introduction of LP/intrinsic	LA: unsaturated B atoms	electrocatalytic nitrogen reduction to ammonia	^14^N_2_/^15^N_2_ exchange experiment, DFT
		LB: unsaturated N atoms		
nitrogen- and boron-incorporated graphite carbon materials^66^	introduction of LP	LA: electron-deficient B atoms	hydrogenation	DFT
		LB: electron-rich N atoms		
TM- and boron-codoped black phosphorus^67^	introduction of LP	LA: transition metal	N_2_ electrochemical reduction	DFT
		LB: B atoms		
NU-1000,^68,69^ MIL-101(Cr)^31,70−72^	introduction of LP	LA: Lewis acidic organics anchored into MOF	hydrogenation	FTIR, NMR, DFT
		LB: Lewis basic organics anchored into MOF		

Unlike homogeneous FLPs, which are structurally and mechanistically solved by NMR studies, (single-crystal) X-ray diffraction (XRD), and photoluminescence (PL) spectroscopy^44^ supported by DFT calculations, the identification of heterogeneous FLP catalysts requires more vigorous procedures, which may include the use of multiple advanced techniques. Meanwhile, to complete the mechanistic studies between adsorbates and active centers of catalysts, the crystal structure, nature of the active sites, variations in charge distribution of bonds, and molecular dynamics of both support and reactive intermediates need to be demonstrated. In homogeneous systems, identified FLP sites are the contributor to molecule activation, while in heterogeneous systems, surface electronic and geometric properties impose additional adsorbate–support interactions that are often underexplored. The reaction environment also complicates the studies. Limited by the time resolution of characterization techniques, there is not yet a reliable direct way except computational simulations,^34,45^ which give ideas on feasible transition states (TSs). Additionally, the debate about how small molecules are activated by the FLP mechanism constantly prevails. In homogeneous FLPs, most of the activation mechanisms are concluded to involve heterolytic cleavage of bonds, driven by ET and/or electric field (EF),^34,46,47^ while homolytic cracking involving single electron transfer (SET) and frustrated radical intermediates cannot be ruled out.^19,44,48−50^ In heterogeneous FLP, heterolytic cleavage is commonly assessed as the dominant mechanism.^34^ While good reviews on these cleavage mechanisms have been reported,^34,47^ they will not be reiterated here.

This Account will summarize the combination of techniques used to effectively characterize heterogeneous FLP sites and adsorbate–support interactions, addressing a key research gap. More importantly, it emphasizes the significance of intrinsic catalyst properties and reaction conditions in manipulating catalytic behaviors and illustrating overall reaction mechanisms in the concept of FLP chemistry. Some well-rounded examples are presented to support these new research perspectives. Notably, the systems we studied differ significantly from conventional solid FLPs due to the electronic properties of the supports. Each section elaborates on these differences.

## 2 Induced FLP Active Sites in Zeolite and MOF Materials

In heterogeneous catalysts, most reported FLP systems feature unquenched Lewis acid and base sites, which are generated by molecule immobilization (semisolid FLPs), through intrinsic atomic arrangements or via postsynthesis in rigid lattice topography (solid FLPs).^73^ In contrast to some homogeneous LA–LB pairs, which are connected by polar bonds and can still activate small molecules, quenched FLP chemistry is rarely considered in the undistorted moiety of solids. This limitation arises from the stronger metallic or covalent bonds that connect atoms in extended lattices coupled with minimal electronegativity differences, which result in reduced polarization. However, our recent studies have demonstrated the feasibility of inducing LA and LB sites in zeolites^1,74^ and metal–organic frameworks (MOFs)^2^ through external polar species and stimulus (i.e., light), respectively. In both cases, the dative bonding or polarity between two sites plays a crucial role in facilitating these interactions.

### 2.1 Induced FLPs by Polar Adsorbates in SAPO Zeolites

Zeolites are commercially explored catalysts in the petroleum industry, pollution treatment sector, and other applications.^75^ Composed of TO_4_ tetrahedra from main-group elements (T = Si, Al, P), their Brønsted acid sites (BASs) arise from hydroxyl protons in the Al–O(H)–Si framework, balancing the charge difference created by the incorporation of a low-covalent element according to the principle of electroneutrality. In addition to protons, extraframework alkali cations are also exchanged in zeolites for charge balancing. The Lewis acid site (LAS) originates from framework-associated aluminum, extraframework aluminum (EFAl) species formed during synthesis or in steam-assisted treatments, and other extraframework metal species.^76^ Altering starting materials, structure-directing agents, and synthesis methods allows control of crystal structures with varied pore shapes, particle sizes, morphology, and acidity for enhanced catalytic performances.^77^ Simultaneously, the flexibility of the zeolite framework enables “elasticity” in bond distances and angles under pressure or heat or during gas adsorption and diffusion.^78^

In silicoaluminophosphate (SAPO) zeolites, regardless of crystallography, the presence of two heteroatoms weakens the bond strength by increasing orbital mismatch, resulting in a more fragile Al–O bond in the SiO(H)→Al moiety. Our studies have shown that the adsorption of polar gaseous molecules, such as acetone, methanol, and water, disrupts this dative bond and generates FLP active sites.^1^ The electron-rich oxygen from the hydroxyl of BAS acts as an LB, while the adjacent electron-deficient aluminum serves as an LA in this system. This concept, *proposed for the first time*, was verified experimentally by combined solid-state nuclear magnetic resonance spectroscopy (ssNMR), diffuse-reflectance infrared Fourier transform spectroscopy (DRIFTS), and structure refinement techniques.

**Figure 2:**
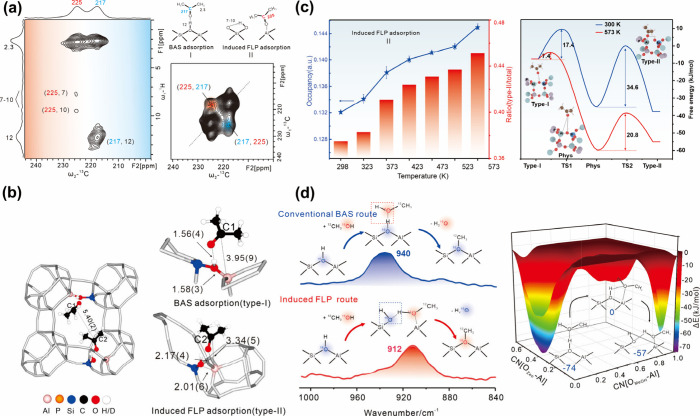
(a) 2D ^13^C–^1^H HETCOR ssNMR spectrum of ^13^C-2-acetone adsorbed SAPO34 showing correlations between adsorbates and framework protons and the 2D ^13^C–^13^C PDSD NMR spectrum to prove the spatial proximity between BAS-type (mode I) and FLP-type (mode II) adsorption of acetone. (b) Crystallography models from Rietveld refinement of SXRD patterns to visually illustrate two adsorption modes of acetone in SAPO34. (c) Increasing trend of induced FLP occupancy with increasing temperature, fitted from Rietveld refinement of variable-temperature SXRD patterns of acetone-adsorbed SAPO34, together with the potential energy surfaces of the transformation from BAS to induced FLP by acetone adsorption at 300 and 573 K. (d) Schematic diagram of the formation of surface methoxyl species via the traditional BAS route and proposed induced FLP route, together with the calculated energy landscape supporting the transformation of adsorped methanol on BAS to become induced FLP. Panels (a), (b), and (d) reproduced from ref (1). Copyright 2021 American Chemical Society. Panel (c) reproduced from ref (74). CC BY 4.0.

Acetone is the typical probe molecule for determining acid strength in zeolites.^79−83^ It forms hydrogen bonds with bridging-hydroxyl protons of BASs and exhibits an O–Al interaction with LASs, leading to downfield shifts of carbonyl signals. In the ^13^C magic-angle spinning (MAS) ssNMR spectrum of ^13^C-2-acetone-adsorbed SAPO34, two peaks were observed in the carbonyl region. The peak at 217 ppm (Type I) was assigned to acetone interacting with BAS according to the literature.^84,85^ The other peak at 225 ppm (Type II) disappeared after rehydration, indicating its contribution from LAS. This peak exhibited a different interaction mechanism compared to that of BAS and was later confirmed to arise from acetone adsorption via an induced LB–LA pair. Additionally, a 2D ^13^C–^1^H heteronuclear correlation (HETCOR) NMR spectrum was measured (Figure 2a), revealing a correlation peak at (225, 7–10) ppm, which indicated the proximity of the LAS to the hydroxyl group. Furthermore, the 2D ^13^C–^13^C proton-driven spin diffusion (PDSD) NMR experiment (Figure 2a) offered additional evidence for the spatial proximity of acetone molecules in two modes (their distance turned out to be 5.40(2) Å). By refining synchrotron X-ray diffraction (SXRD) and neutron powder diffraction (NPD) data, framework geometries, atomic occupancies, and, more importantly, the two forms of adsorption mode were visualized (Figure 2b). Furthermore, in the in situ SXRD study, we found that the occupancy of induced FLPs increased with rising temperature. This finding suggests that elevated temperatures can facilitate the transformation of BASs to induced FLPs. The lower transformation energy between BASs and induced FLPs at higher temperatures than that at low temperatures was also verified by theoretical calculations (Figure 2c).

With polar adsorbates, the framework reconstruction and dynamics of SAPO materials extended their applications as metal-free thermocatalysts. The commercial methanol-to-olefin (MTO) reaction^86,87^ was reviewed, and studies on the adsorption mechanisms of methanol on pristine SAPO zeolite further solidified that FLPs can be induced by active adsorbates.^1^ Similar to acetone adsorption, measurements proved the spatial proximity of carbon species, elongated Al–O bonds, and compressed Al–O–Si bond angles. Additionally, the observation of surface methoxy species in the methanol–adsorbed ^13^C MAS ssNMR spectrum measured at room temperature suggested an unusual reaction pathway. The in situ DRIFTS experiments using ^18^O/^16^O-labeled methanol revealed that the formation of surface methoxy species from methanol is catalyzed via the induced FLP route rather than the traditional BAS route. As shown in Figure 2d, when using nonlabeled methanol, the peak assigned to the ^12^C–^16^O stretching vibration in methoxy species appeared at 940 cm^–1^, while it shifted to 912 cm^–1^ (^12^C–^18^O) with ^18^O-methanol. This shift indicates that the methoxy oxygen originates from methanol, confirming that there is no C–O bond cleavage during the reaction. This finding contrasts with the conventional BAS route, which likely involves C–O cleavage of methanol to eliminate H_2_O. The calculated energy landscape of methanol adsorption and activation (Figure 2d) further extended and supported the possibility of the transformation of BASs into induced FLP sites.

Assisted by the polarity of adsorbates, the FLP system in zeolitic materials with weaker bonding due to insufficient dative orbital overlap will be induced, particularly in the regions of steric constraints of high strain (i.e., channel interfaces). In SAPO materials, separated Si–O(H) and Al create new active sites that offer alternative reaction pathways with a lower activation energy. To effectively track bond breakage and the formation of induced FLPs, a combination of ssNMR, SXRD, NPD, and DRIFTS is essential for characterizing zeotype materials. Drawing insights from the MTO reaction, we anticipate broader applications of SAPO zeolites designed with induced FLP chemistry.

### 2.2 Photoinduced FLP in Ru/UiO-67-bpydc

Another class of crystalline porous materials, MOFs, consist of metal-containing nodes connected by organic linkers and feature both Lewis acidity and Lewis basicity.^88,89^ By variation of the length and composition of linkers, the pore sizes of materials and the nature of active sites can be adjusted. One of the main challenges facing MOFs is structural instability. Addressing this during synthesis requires careful selection of high-temperature-resistant linkers with appropriate topology, precise concentration control, and the use of suitable solvents. Alternatively, postsynthetic modification offers a flexible approach, targeting metal nodes, linkers, or guest molecules to enhance stability and performance.^88,90^ Selecting linkers that possess aromaticity or π-conjugated systems, in conjunction with appropriate metal ions, enables the materials to exhibit optical activity or photoluminescence upon irradiation.^91,92^ Consequently, MOFs hold significant potential for a variety of applications, including catalysis, gas storage and separation, fuel cells, and luminescent sensors.

Universitet-i-Oslo (UiO) series MOFs are based on the Zr_6_O_4_(OH)_4_ node and have exceptional chemical stability in strongly acidic conditions due to the high coordination number of Zr^IV^.^93^ Among various UiO-type MOFs, UiO-66-NH_2_ and UiO-67-bpydc are isostructural, with the former using 1,4-benzenedicarboxylate (BDC) as the linker and the latter obtained with the longer 4,4′-biphenyldicarboxylate (BPDC) as the linker. Both frameworks were investigated for hydrogen storage, with UiO-67-bpydc showing a hydrogen storage capacity that is double that of UiO-66-NH_2_, likely attributable to its larger surface area and greater concentration of active sites.^94^ Recently, we examined their hydrogen dissociation capability and found that the activity of Ru-modified UiO-67-bpydc is promising. Under light illumination, the Ru–N bond is polarized, leading to metal-to-ligand charge transfer (MLCT). This interaction facilitates Ru^+^–N^–^-mediated FLP chemistry, enhancing the framework’s ability to activate small molecules effectively.

**Figure 3:**
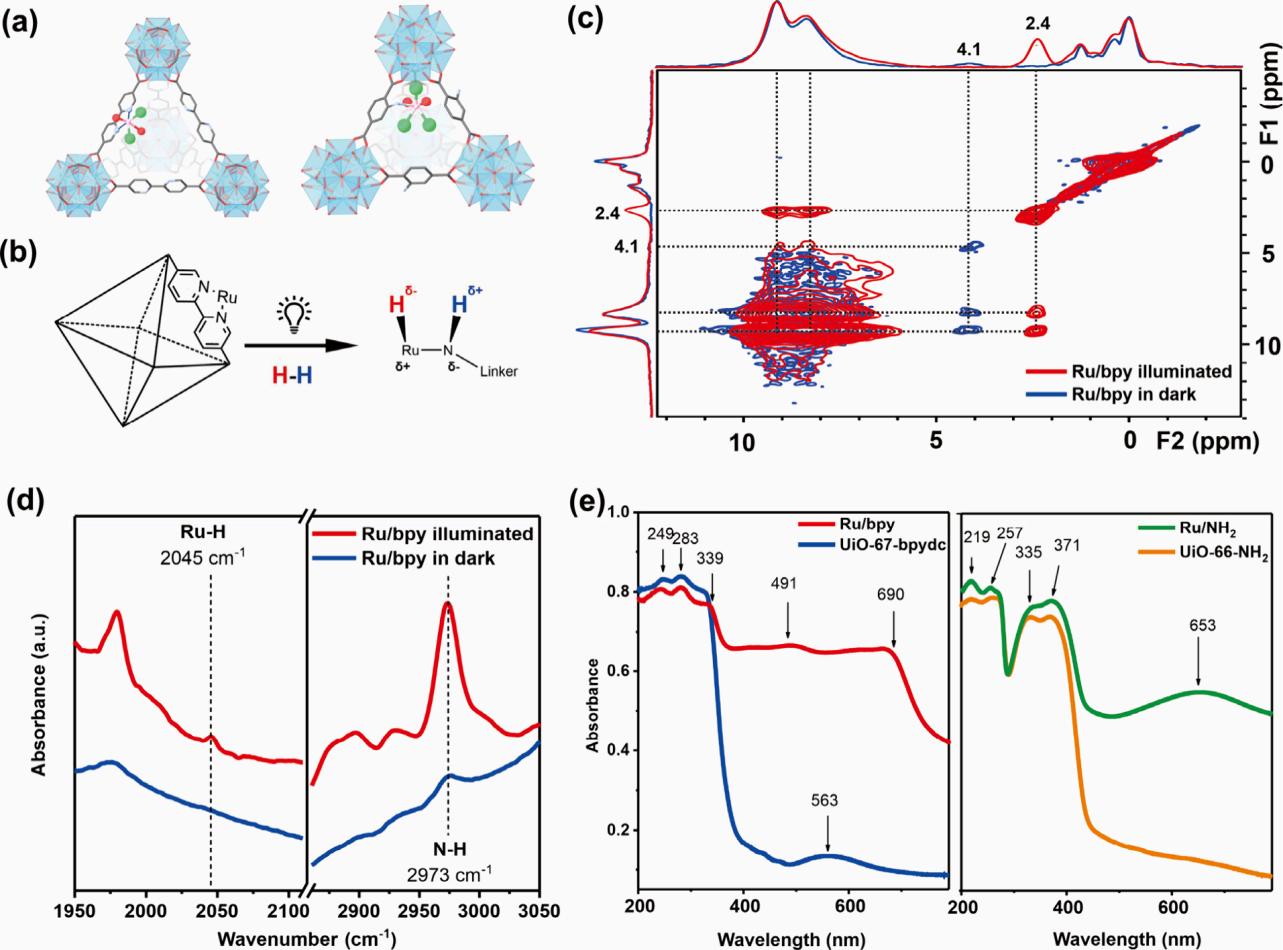
(a) Structure diagrams of Ru/bpy (left) and Ru/NH_2_ (right) that reveal coordination environments of two materials, with information obtained from EXAFS spectra fitting. (b) Schematic depiction of the hydrogen dissociation mechanism in the Ru/bpy FLP system. (c) 2D ^1^H–^1^H COSY NMR spectrum of H_2_-adsorbed Ru/bpy before and after illumination. (d) In situ FTIR measurement showing the appearance of the Ru–H bond and sharper N–H bond upon light illumination, supporting hydrogen dissociation. (e) UV–vis spectra of two UiO-type MOFs and corresponding Ru-modified materials, suggesting MLCT in Ru/bpy and therefore stronger polarization of the Ru–N bond. Reproduced from ref (2). CC BY 4.0.

As shown by Ru K-edge extended X-ray absorption fine structure (EXAFS), the main difference between the two UiO types of sixfold-coordination environment was the connectivity of N-containing ligands (i.e., bidentate and monodentate), as shown in Figure 3a, of which Ru/UiO-67-bpydc (Ru/bpy) has two Ru–N bonds from the bpy moiety while Ru/UiO-66-NH_2_ (Ru/NH_2_) has one Ru–N bond per benzene. As a result of differences in metal–ligand geometry and orbital overlap, these two catalytic materials showed distinct activities in a hydrogen–deuterium (H–D) exchange experiment. Ru/bpy achieved at least 5-fold increase in the amount of HD formed under illumination (Figure 3b), while Ru/NH_2_ remained inactive. The hydrogen storage ability of Ru/bpy in the dark was proved by ^1^H ssNMR for the first time by showing trapped gas-phase hydrogen at 4.1 ppm. After illumination, an additional peak at 2.4 ppm for NH^+^ appeared, which was in close proximity to protons from bipyridine (7–9 ppm) in the 2D ^1^H–^1^H correlation spectroscopy (COSY) ssNMR spectrum (Figure 3c). Supplementary operando information was provided by in situ DRIFTS, as shown in Figure 3d. The appearance of Ru–H stretching at 2045 cm^–1^ and stronger N–H stretching at 2973 cm^–1^ when Ru/bpy was irradiated confirmed heterolytic cleavage of hydrogen by the Ru^+^–N^–^ FLP. The weak Ru–H peak indicated some extent of proton migration, but this was undetectable in ssNMR.

To justify the hydrogen activation via FLP induction under light illumination, we performed a range of optical characterizations. As shown by Figure 3e, three characteristic peaks for MLCT appeared at 339, 491, and 690 nm in UV–vis spectra of Ru/bpy. Meanwhile, the peak at 563 nm corresponding to linker–linker charge transfer (LLCT) disappeared in comparison to pristine UiO-67-bpydc. PL and time-resolved photoluminescence (TRPL) measurements further suggested that the introduction of Ru offered a lower-energy pathway that mitigated LLCT, with the short exciton lifetime indicating the relaxation pathway via MLCT. In comparison, the UV–vis spectrum of Ru/NH_2_ had a new rising peak at 653 nm for a weak Ru d–d transition only, while other peaks for π–π* excitation at linkers and LLCT (between N lone pair and linker π*) remained. Both PL and TRPL showed that Ru immobilization strengthened the dominant LLCT in Ru/NH_2_, leading to slower charge recombination and a longer lifetime of excitons.

The incorporation of ruthenium into UiO-67-bpydc facilitates the formation of ruthenium complexes with a bidentate organic linker, establishing a strong pathway for electron delocalization. Light irradiation pushed a stronger polarization in the Ru–N bond via MLCT, resulting in the formation of the Ru^+^–N^–^ FLP, which heterolytically cleaves hydrogen gas molecules. The direct evidence for H_2_ dissociation at the active sites was obtained by 1D ^1^H and 2D ^1^H–^1^H ssNMR, and DRIFTS measurements. UV–vis, PL, and TRPL are essential techniques to illustrate the charge transfer mechanisms in photocatalysts. Inspired by this system, the application of a metal-modified MOF in photocatalytic activation for small molecules is an intriguing direction to explore.

## 3 Ru–O FLP and Hydrogen Spillover Effect

In the structure of heterogeneous catalysts, FLP chemistry can be activated through electron redistribution by external stimuli, facilitating gas adsorption and small-molecule cracking. Apart from this, effective catalysis also requires the design of catalytically active sites for dynamic product formation. The introduction of dopants into unreactive supports has been shown to effectively modify surface electronic properties and acidity,^95−97^ which motivated our research on the incorporation of transition metals (TMs). Among M–X systems, we justified that the isolated Ru atoms, which are stabilized by oxygen atoms of the support, form the Ru–O FLP system for small-molecule activation.^3,98−101^ This is different from the classic solid FLPs formed between metal clusters or NPs and oxygen from the support. Furthermore, due to the inherent redox properties, ruthenium not only participates in FLP chemistry but also exhibits a hydrogen spillover effect (HSPE), which helps prevent hydrogen poisoning of metal active sites, thereby enhancing the overall catalytic performance.

### 3.1 Hydrogen Spillover Effect

The hydrogen spillover effect refers to the migration of dissociated hydrogen atoms in H^+^–e^–^ pairs over surface active sites in materials. This phenomenon was first reported by Khoobiar et al. in 1964 in reducible metal oxides, WO_3_ mixed with Pt/Al_2_O_3_.^102^ Subsequent discoveries also identified this effect in supports like CeO_2_ and TiO_2_.^103^ In these reducible systems, the additional electron is accommodated by reduction of the adjacent metal, which thermodynamically drives the migration of protons and their attachment to oxygen species.^103−105^ Changes in oxidation state can be detected by X-ray photoelectron spectroscopy (XPS) and EXAFS, and the formation of OH, OH_2_, and defective oxygen species can be traced using DRIFTS and ^1^H ssNMR. The hydrogen spillover shifts the hydrogen dissociation equilibrium to the right, playing a crucial role in reducing metal poisoning. This ensures that a certain number of accessible and preferred metal adsorption sites remain available for reactants rather than hydrogen atoms, thereby facilitating dynamic interactions. Materials exhibiting this effect can be recovered after exposure to an oxygen-rich or inert environment. Notably, defective nonreducible metal oxides that are chemically bound to unsaturated organic molecules also have hydrogen spillover ability, with organics consuming dissociated hydrogen (or protons) to mitigate poisoning.^103,106^ Examples of such systems include metal-modified γ-alumina, silica, and metal core–shell zeolites that catalyze hydrogenation reactions.^106^ However, the precise pathway of hydrogen or proton diffusion remains uncertain, as H–D isotopic labeling experiments cannot definitively distinguish between diffusion and exchange processes.

### 3.2 FLPs on Polar Surfaces

**Figure 4:**
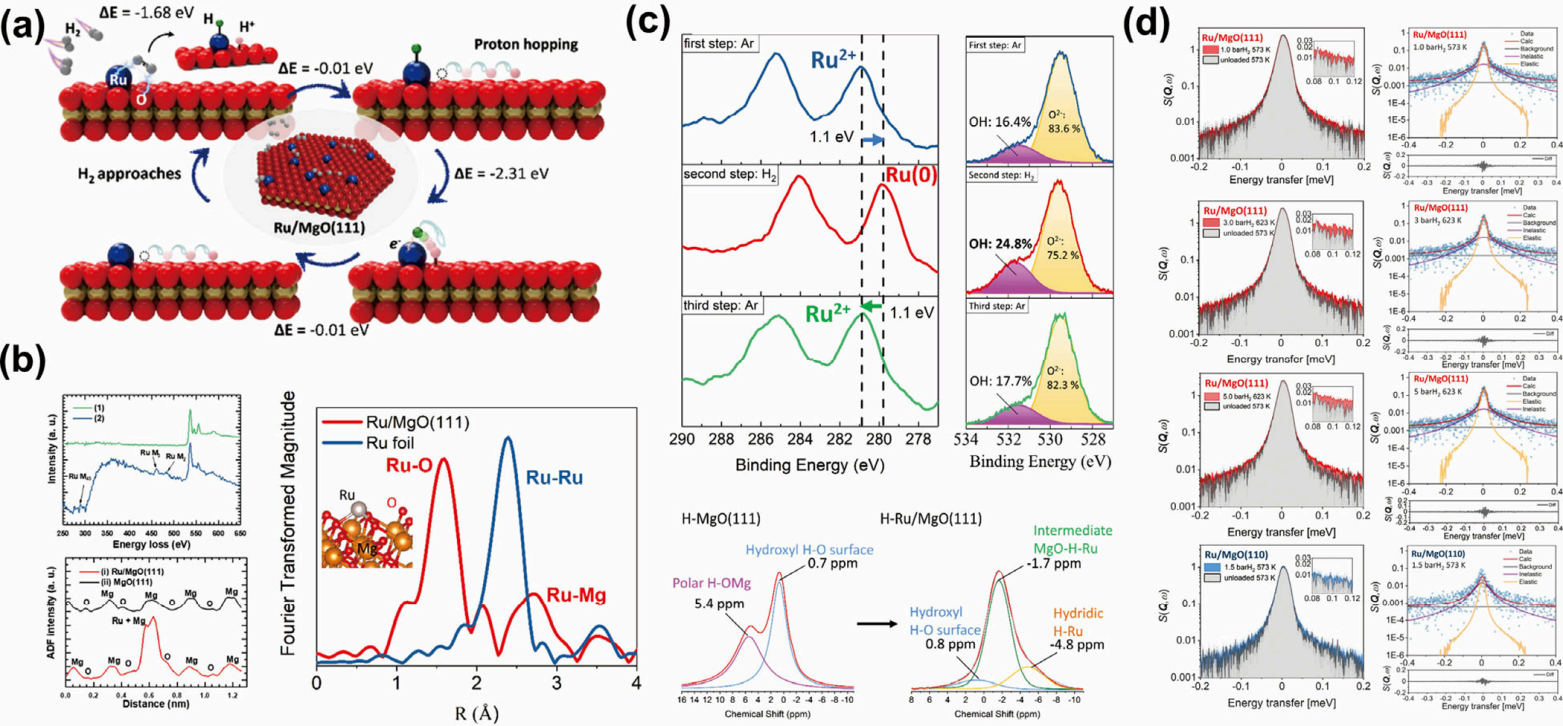
(a) Graphic illustration of hydrogen dissociation mechanism on Ru/MgO(111) surface via Ru^2+^–O^f^ FLP sites and hydrogen spillover from Ru ions to the oxide anions of the support. (b) EELS spectra extracted on oxygen atoms showing both oxygen and ruthenium signals and atomic distributions acquired along the line based on HAADF-STEM image, and EXAFS spectra of the Ru/MgO(111) sample measured at the Ru K-edge, evidencing the atomic locations of Ru. (c) In situ XPS spectra presenting the redox ability of Ru^2+^ ions (extraction of e^–^ from H^+^–e^–^ pair) in the Ru/MgO(111) lattice, which assists hydrogen dissociation via the hydrogen spillover effect, and ex situ ^1^H NMR spectra showing Ru–H and Mg–O(H)–Ru species to support the FLP-type hydrogen activation mechanism. (d) Fitted QENS spectra of MgO(111) and MgO(110) at momentum transfer *Q* = 0.89 Å, with broadening in peaks of the MgO(111) sample attributed to the H^+^ diffusion. Panels (a–c) reproduced from ref (3) Copyright 2021 American Chemical Society. Panel (d) reproduced from ref (101). CC BY 4.0.

Although both FLP chemistry and hydrogen spillover effects are well-documented in the field of catalysis and contribute to reaction conversion, the interplay between these two phenomena is seldom explored in reaction processes. Herein we present an example of metal-modified magnesium oxide with outstanding surface properties. The polar O-terminated (111) facet of Cs-doped Ru/MgO has enhanced turnover frequencies of ammonia synthesis compared to other nonpolar facets (110) and (100), via both FLP active sites and hydrogen spillover.^3,98,101^ A new spillover mechanism for polar nonreducible oxides is proposed (Figure 4a).

Three different ruthenium-doped MgO materials with exposed (111), (110), and (100) facets were synthesized. Compared to other nonpolar facets, no significant defects were observed in transmission electron microscopy (TEM) images of Ru/MgO(111). In this system, due to strong metal–support interaction and high dispersion, the ruthenium atom was located above magnesium atoms rather than being incorporated into the lattice to form metallic motifs, as seen in electron energy loss spectroscopy (EELS) and high-angle annular dark-field scanning transmission electron microscopy (HAADF-STEM) in Figure 4b. Least-squares fitting of EXAFS of the sample (degassed and reduced) showed the presence of Ru–O bonds and the absence of Ru–Ru bonds (Figure 4b), confirming the formation of framework Ru cations coordinated with three adjacent oxygen (Ru-OOO sites). The oxidation state of ruthenium was supported by XPS measurements. These Ru cations are stabilized by the electrostatic interaction and surface polarity of the terminated-oxygen facet via charge transfer. Hydrogen dissociation was catalyzed by Ru/MgO(111), where the formation of Ru–H bonds on ruthenium cations and O–H bonds on isolated bridging O^2–^ sites was observed by ^1^H ssNMR (Figure 4c), Fourier transform IR spectroscopy (FTIR), and (O 1s) XPS. These findings supported the formation of the Ru^2+^–O^f^ unquenched FLP pair with an O^f^ siting adjacent to Ru-OOO sites, which acted as the active site for small-molecule activation.

Simultaneously, two abnormal phenomena related to hydrogen spillover effects were observed during operando characterizations. In situ XPS and X-ray absorption near edge spectroscopy (XANES) measurements revealed reversible changes in the oxidation state of ruthenium (Figure 4c). Since the sample had been treated under hydrogen before reaction, this further reduction in oxidation state during the reaction was mysterious. Additionally, the positive hydrogen order and negative hydrogen retardation order in Ru/MgO(111) suggested effective hydrogen removal rates and less significant intermediate species on metal sites, which was attributed to high activity.^98^ It was later proven by quasi-elastic neutron scattering (QENS) (Figure 4d) that the electron acceptance at ruthenium sites facilitated the H^+^ migration on Ru/MgO(111). Instead of unreacted hydrogen molecules or dissociated hydridic species migrating across the surface, QENS provided strong evidence for the formation and diffusion of protonic species.^3,101^ The fitted proton diffusion distance corresponded closely to the atomic spacing between oxide anions on MgO(111), with a diffusion rate of (1.2–3.1) × 10^–5^ cm^2^/s, comparable to known H^+^-conducting materials. As further supported by DFT calculations, this proton diffusion was enhanced by the local electric field and surface polarity of MgO(111), with the highest proton adsorption energy at the bridging oxygen sites. Combining all the information, protonic species diffuse/hop over the O-terminated MgO(111) surface assisted by the reduction of ruthenium cations, which prevents hydrogen poisoning of ruthenium species. Up to this point, the bifunctional behaviors of ruthenium dopants have been clearly revealed.

Atomic arrangements determine the surface electronic properties, such as polarity, which influence metal–support interaction and product selectivity, based on prior studies.^107,108^ In the case of polar oxygen-terminated MgO(111), the polarity from unique atomic arrangements with alternating layers of Mg^2+^ cation and O^2–^ anion and the defect-free surface bestowed its capability to have both hydrogen spillover ability and FLP chemistry when doped with ruthenium. Effective charge transfer on this polar surface immobilized Ru^2+^ cations above Mg^2+^ cations and formed the Ru^2+^–O^f^ FLP that can heterolytically cleave H_2_. Following H_2_ dissociation and formation of a Ru–H bond, Ru(II) retained electrons and detached protons, which propagated for a distance via oxide anion species, preventing metal poisoning. The ammonia cracking activity of Ru/MgO(111) was also studied in the subsequent year, achieving a remarkable conversion of 98% at 425 °C on the polar Ru/MgO(111) surface.^99^ During investigations, characterization techniques such as TEM, FTIR, in situ XPS, and in situ X-ray absorption spectroscopy (XAS) are indispensable for understanding surface properties and identifying active site environments. Meanwhile, fitting QENS spectra provided valuable insights into diffusion species and mechanisms and quantified diffusion parameters, supporting our understanding of hydrogen spillover dynamics.

### 3.3 FLPs on Nonpolar Surfaces

**Figure 5:**
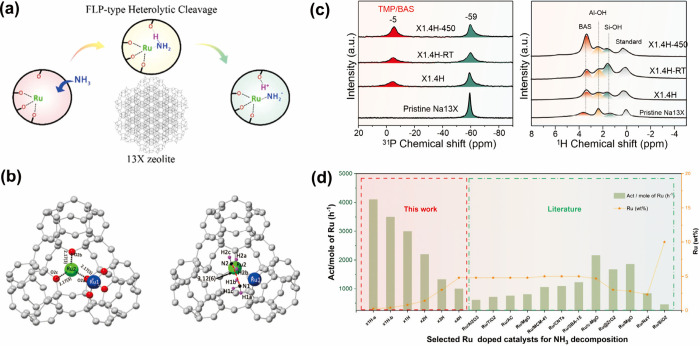
(a) Graphical illustration of the Ru–O FLP chemistry for heterolytic ammonia cracking in Ru/13X zeolite. (b) Crystallography models from Rietveld refinement of SXRD data to visualize the location of Ru ions residing in pores of 13X. (c) ^31^P NMR spectra of TMP-adsorbed samples and ^1^H MAS NMR spectra of samples (X1.4H represents 1.4 wt % Ru). Both are consistent with the regeneration of BAS via FLP-type cleavage of ammonia at 450 °C. (d) Summary of catalyst activity per Ru wt %, showing more effective decomposition reactions in Ru/13X systems. Reproduced from ref (100). CC BY 4.0.

In 2023, joint chemistry involving FLPs and hydrogen spillover was also proposed in a nonpolar surface, Ru-doped 13X (Ru/13X) (Figure 5a). High conversion rates in ammonia cracking highlighted the importance of this synergistic effect.^100^ Unlike Ru/MgO(111), which requires charge transfer and a local electric field, the BAS in zeolites anchors the ruthenium cation and promotes proton migration. Fitting of EXAFS spectra showed the successful ion exchange of Ru^3+^ cations with framework H^+^ at BASs, as evidenced by coordination with oxygens. High-resolution SXRD and NPD identified two Ru^3+^ sites and confirmed the Ru-OOO lattice (Figure 5b). The heterolytic cleavage of the ammonia molecule by the Ru^3+^–O^f^ FLP was supported by an increase in proton occupancies after the reaction (observed in NPD) and a stronger BAS peak in trimethylphosphorus (TMP)-assisted ^31^P and ^1^H ssNMR (Figure 5c). DFT calculations, along with a previous report on the hydrogen spillover capability of zeolites,^109^ validated the reduction of a metal cation and migration of a proton to the zeolite framework. This system offered insights into the role of the ruthenium “single atom” in the zeolitic framework, which assisted ammonia decomposition (Figure 5d). Future work, such as in situ X-ray-related spectra and FTIR, is needed to provide more experimental proof for hydrogen migration.

## 4 TM–X FLP and H_2_O–H_2_-Assisted Reactions

As discussed, the relationship between optimal water coverage on the Al_2_O_3_(110) facet and the dissociation ability of the Al^III^–O_3b_ pair highlights the critical role of water in activating catalytic sites. In certain metal-doped FLP systems, we observed that supplying H_2_–H_2_O under operando conditions offers both activity and product selectivity for the synthesis of organic compounds at newly designated sites.^4,110^

### 4.1 Acid Activity in the Co–NC System

**Figure 6:**
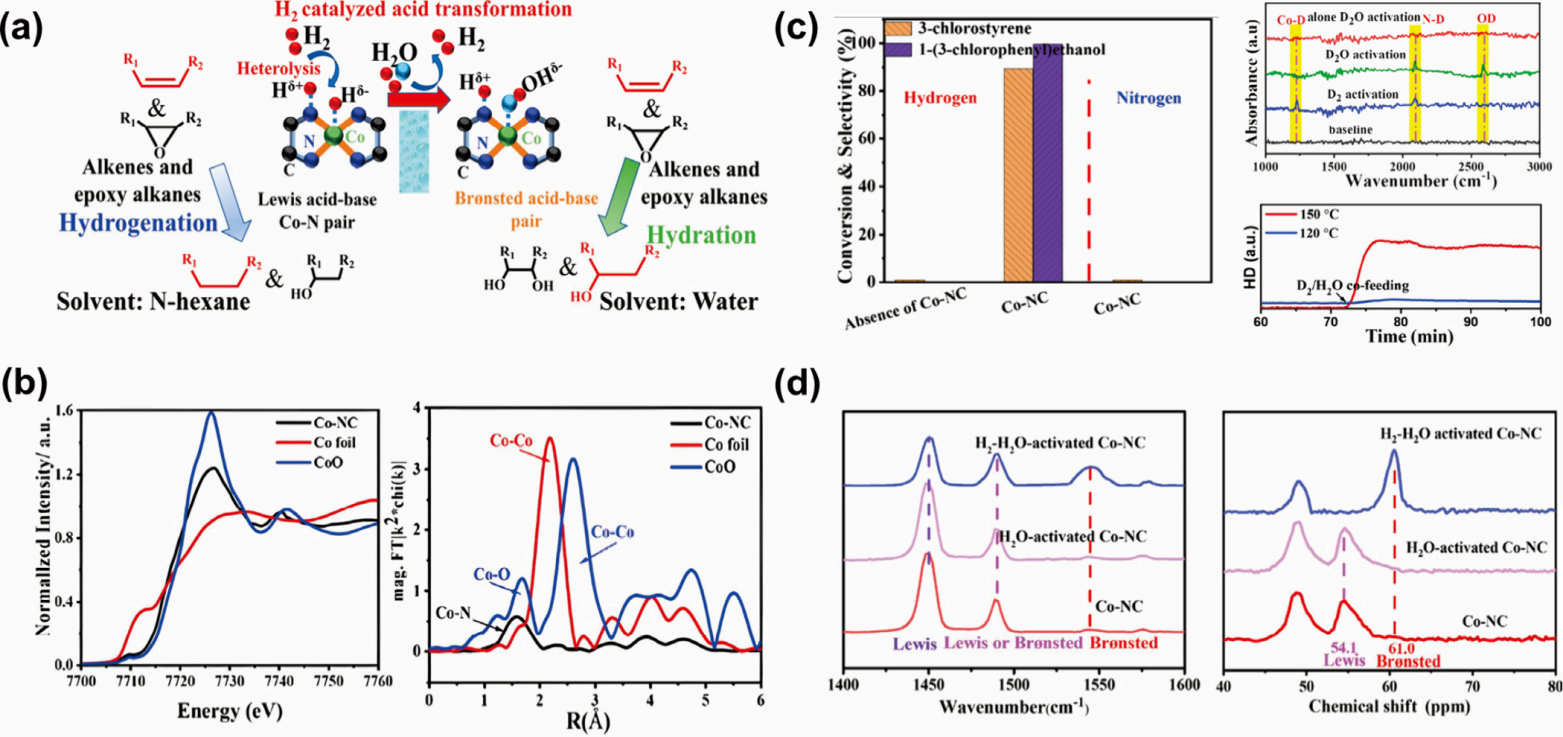
(a) Proposed reaction pathway for the hydration reaction of alkenes and epoxy alkanes, via FLP active sites. Co–H formed from H_2_ cracking catalyzed the acid transformation. (b) XAS (XANES and EXAFS) spectra revealing the coordination environment of single-atom Co. (c) Conversion comparison between reactions in different conditions, showing 99.5% selectivity for the formation of 1-(3-chlorophenyl)ethanol in water solvent under H_2_ flow, and ATR-IR measurements under D_2_/D_2_O conditions, showing the operando formation of active sites. (d) (left) In situ DRIFTS spectra and (right) ^31^P ssNMR spectra of TMPO-adsorbed Co–NC. Both plots revealed the effective formation of BASs in the Co–NC catalyst under H_2_–H_2_O environment only. Reproduced from ref (4). Copyright 2021 American Chemical Society.

Hydration of alkenes and epoxy alkanes is an acid-catalyzed reaction that requires high stereoselectivity for the desired products. Most studies focused on transition metal complex-based homogeneous catalysts with oxygenation and reducing reagents to minimize the use of strong acids.^111^ Cobalt complexes emerged as promising candidates for hydrogen transfer reactions.^112^ However, challenges such as metal contamination and the complexities of liquid separation hinder the recovery of both products and homogeneous catalysts. In 2021 a novel heterogeneous system—single-atom Co-dispersed nitrogen-doped carbon (Co–NC)—was reported, achieving over 99% selectivity for products.^4^ Under the assistance of the Co–N FLP on the catalyst surface, H_2_ is coactivated to form Co–H and N–H in a similar way to those in Ru–O FLP systems. However, the larger difference in electronegativity between Co and H (Co, 1.88; H, 2.20) compared to Ru–H (Ru, 2.20) gives a stronger hydride character (H^δ−^), making the more polar Co–H bond intolerant to water molecules (H^δ+^–OH^δ−^). Water regenerates gaseous H_2_ and concomitantly creates a Co–OH surface with acidic N–H synergetic pair sites. They can then selectively hydrate the organics to products via an acid-catalyzed mechanism following Markovnikov’s rule (Figure 6a).

Inductively coupled plasma optical emission spectrometry (ICP-OES), XRD, TEM with energy-dispersive spectroscopy, and Co K-edge XANES were used to visualize the structure of the catalyst. As shown in Figure 6b, no Co–Co bonding was detected in EXAFS, and therefore, no clusters or nanoparticles were formed. Instead, cobalt was coordinated to four nitrogen ligands with the nitrogen-doped carbon (NC) support. Charge transfer from cobalt to pyridinic nitrogen indicated the presence of Lewis acidic cobalt with an oxidation state between 0 and +2 and Lewis basic nitrogen (from N 1s and Co 2p XPS). Under H_2_–H_2_O conditions, Co–NC was highly effective in catalyzing the hydration of alkenes to alcohols and the conversion of epoxy alkanes to diols. Control experiments showed the necessity of H_2_ gas, H_2_O, and Co–NC for high activity and selectivity (Figure 6c), which was related to an increase in material acidity, supported by pyridine DRIFTS and ^31^P ssNMR spectra, as shown in Figure 6d. The detailed mechanism, explaining how H_2_–H_2_O monitored system acidity and basicity and catalyzed the reaction, was studied by in situ attenuated total reflection infrared spectroscopy (ATR-IR) (Figure 6c), ^1^H ssNMR, and ex situ XPS. After introduction of D_2_ gas, Co–D^δ−^ and N–D^δ+^ stretching vibrations were directly detected. When D_2_O was introduced, a peak at 2581 cm^–1^ for Co–OD^δ−^ appeared, while a decrease in the peak for Co–D^δ−^ occurred. Proton exchange did not occur at the N–H^δ+^ site. Instead, H^δ+^ from H_2_O coupled with Co–H^δ−^ and released H_2_, with the remaining OH^–^ electrostatically attracted to cobalt. Meanwhile, an increase in binding energy was shown in N 1s XPS as pyridinic N donated electrons to the proton; on the contrary, the Co 2p XPS peak shifted to a lower binding energy since cobalt attracted electrons from the hydroxyl group.

### 4.2 Dual Active Sites in Partially Oxidized MAX Phase

The bifunctionality of partially oxidized MAX phase Pd/Ti_3_AlC_2_ for furfural conversion to linear ketones also arises from the formation of a hydrogen FLP (H^δ−^–Pd/TiOH^δ+^) through water-assisted hydrogen spillover.^110^ This system enables asymmetric hydrogenation of the C=O moiety while also providing BAS for the ring opening of furfurals, contributing to their high catalytic performances.

**Figure 7:**
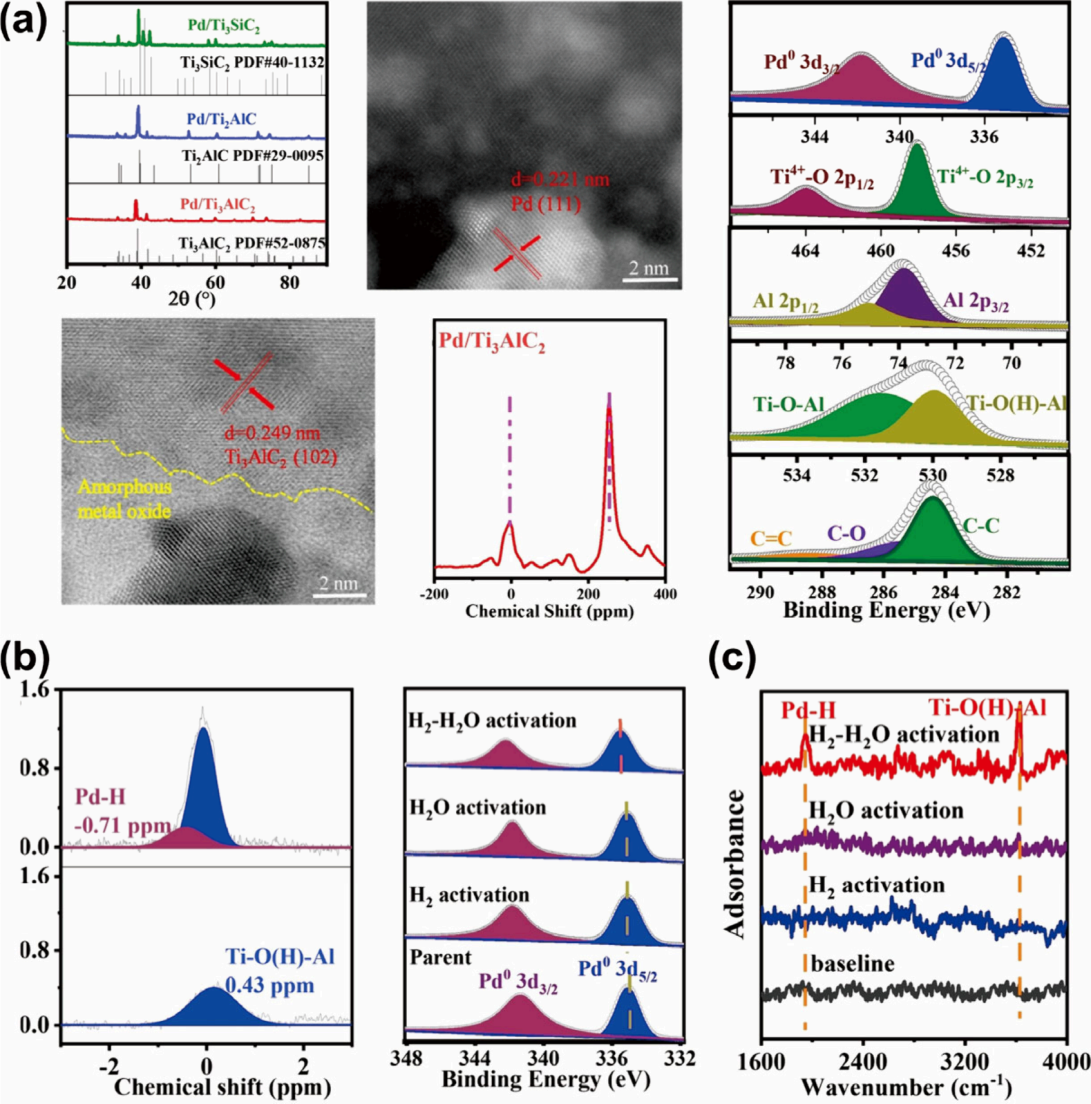
(a) Summary of the XRD patterns, TEM images, ^27^Al MAS spectra, and XPS spectra of Pd/Ti_3_AlC_2_. (b) (left) ^1^H NMR spectra with a new peak for Pd–H observed and (right) in situ XPS spectra with red-shifted peaks. Both showed changes in the Pd coordination environment under H_2_–H_2_O activation. (c) In situ IR spectra for the H_2_, H_2_O, and H_2_–H_2_O activated Pd/Ti_3_AlC_2_ at 90 °C. Reproduced from ref (110). CC BY-NC 4.0.

Compositions and crystallography of Pd/Ti_3_AlC_2_ were characterized by using ICP-OES, XRD, TEM, and XPS, as shown in Figure 7a. Rather than forming metal single atoms, the Pd dopants exhibited particle sizes of 0.221 nm, which is comparable to that of the MAX phase (0.249 nm), and act as the Lewis acid sites as in classic solid FLPs. After the reaction, significant changes were observed in XPS spectra (Pd 2d, Ti 2d, C 1s, and O 1s) of the material. The partial reduction of Ti^4+^ to Ti^2+^/Ti^0^ and creation of more BASs in the form of Ti–O(H)–Al were consistent with the hydrogen spillover behavior mentioned in section 3. This is in line with TiO_2_ being a known reducible oxide for proton migration. Concurrently, the XPS spectra revealed the oxidation of metallic Pd nanoparticles, suggesting the hydride attachment on Pd from heterolytically cleaved hydrogen. ^1^H ssNMR and ATR-IR spectra (Figure 7b,c) provided more intuitive clues for the FLP-type activation, both showing peaks corresponding to bridging hydroxyl Ti–O(H)–Al and oxidized palladium Pd–H. Control experiments and DFT calculations confirmed that water was crucial for promoting the adsorption energy of hydride during hydrogen dissociation on Pd. The stable hydrogen FLP formed Pd–H and Ti–O(H)–Al, thus providing dual active sites for organic synthesis. The success of M–X FLP systems in organic synthesis underscores the ability to create bifunctional catalytic activity under H_2_–H_2_O conditions. These findings prompt further investigation into the influence of metal dopants on surface electronic properties and their role in catalytic processes.

## 5 Conclusions and Perspectives

The pursuit of higher catalytic conversions and selectivity through strategic surface modifications has spurred research into heterogeneous catalysts utilizing FLP chemistry for small-molecule activation and large-scale organic chemical synthesis. This Account highlights our efforts in designing heterogeneous FLP active sites by exploiting the electron distribution in overlapping orbitals and leveraging synergistic effects based on the electronic properties of the materials. We also present mechanistic studies, using various examples, to illustrate the effective combinative use of characterization techniques within the catalysis community. We hope that this Account provides valuable insights into the design of FLP catalysts and mechanistic investigations by considering both the intrinsic properties of materials and the influence of external environments.

In addition to separated FLP sites formed in structurally defective oxides, heterogeneous FLPs can also be induced from undistorted polar moieties through external stimuli such as polar adsorbates and irradiation. These two approaches are based on the understanding of electron density and bond strength of the materials. One requires weak bonding interactions due to energy level mismatch, while the other involves MLCT through good orbital overlap. To validate the presence of FLPs during reactions, the combined use of ssNMR, in situ DRIFTS, and XPS provides the most direct experimental evidence. Supporting information about active sites and structural changes can be obtained through SXRD and NPD, enhancing the overall understanding of the catalytic process.

Simultaneously, M–X FLP systems with synergistic effects can be designed through metal incorporation. A thorough analysis of their electronic properties and reaction environments is essential to explain the observed improvements in catalytic performance. We demonstrated the remarkable hydrogen spillover effect that prevents metal poisoning of Ru–O FLPs at the materials interface. Beyond the techniques mentioned earlier, QENS provides valuable information into proton dynamics, offering a more comprehensive mechanistic understanding. In other M–X FLP systems, the operando supply of H_2_–H_2_O reveals unexpected functionalities of active sites and product selectivity. For example, H_2_-assisted acid transformation in the Co–N FLP system is used in hydration reactions, and H_2_O-assisted hydrogen spillover enhances the bifunctionality in the Pd/Ti–O–Al system. Control experiments with XPS and IR measurements further proved the combination effects.

With the in-depth exploration of structures and mechanisms, more catalytic systems are now capable of utilizing FLP chemistry for bond activation. Looking ahead, we foresee significant growth in the exploration of FLP active sites within heterogeneous catalysts, particularly starting with porous materials, due to their versatile synthesis and structural design capabilities. Meanwhile, investigating dual-active-site systems may reveal new reaction pathways and mechanisms unavailable to single-site catalysts, leading to enhanced catalytic performance. This approach allows for the precise tuning of electronic and steric properties to optimize specific reactions. Moreover, dual active sites offer the potential for developing multifunctional catalysts capable of executing sequential or tandem reactions in a single step, thereby boosting process efficiency.^58^ We encourage the research community to delve into these promising areas to unlock innovative breakthroughs in catalysis. To experimentally demonstrate conversion mechanisms in heterogeneous FLP catalysts, the use of in situ techniques is essential, and we particularly recommend combined in situ ssNMR, DRIFTS, XPS, and XAS measurements. However, the enclosed sample environment and magic-angle spinning measurement of ssNMR constrained its integration with other instruments. Therefore, further efforts are needed to develop in situ DRIFTS and XPS instruments that can work simultaneously. In situ DRIFTS provides insights into functional groups and their transformations, while in situ XPS offers detailed information about the elemental composition and oxidation states. Together, they facilitate the identification of active sites and transient intermediates, which are crucial to understanding reaction mechanisms. This comprehensive understanding aids in optimizing and designing more effective FLP catalysts by tailoring the electronic and steric properties of the Lewis acid and base components to improve reaction rates, selectivity, and stability in catalytic applications.
